# Differential susceptibility of naïve, central memory and effector memory T cells to dendritic cell-mediated HIV-1 transmission

**DOI:** 10.1186/1742-4690-3-52

**Published:** 2006-08-17

**Authors:** Fedde Groot, Toni MM van Capel, Joost HN Schuitemaker, Ben Berkhout, Esther C de Jong

**Affiliations:** 1 Dept. of Human Retrovirology, Academic Medical Centre, University of Amsterdam, Amsterdam, The Netherlands; 2 Dept. of Cell Biology and Histology, Academic Medical Centre, University of Amsterdam, Amsterdam, The Netherlands

## Abstract

**Background:**

Dendritic cells (DC) have been proposed to facilitate sexual transmission of HIV-1 by capture of the virus in the mucosa and subsequent transmission to CD4^+ ^T cells. Several T cell subsets can be identified in humans: naïve T cells (T_N_) that initiate an immune response to new antigens, and memory T cells that respond to previously encountered pathogens. The memory T cell pool comprises central memory (T_CM_) and effector memory cells (T_EM_), which are characterized by distinct homing and effector functions. The T_EM _cell subset, which can be further divided into effector Th1 and Th2 cells, has been shown to be the prime target for viral replication after HIV-1 infection, and is abundantly present in mucosal tissues.

**Results:**

We determined the susceptibility of T_N_, T_CM _and T_EM _cells to DC-mediated HIV-1 transmission and found that co-receptor expression on the respective T cell subsets is a decisive factor for transmission. Accordingly, CCR5-using (R5) HIV-1 was most efficiently transmitted to T_EM _cells, and CXCR4-using (X4) HIV-1 was preferentially transmitted to T_N _cells.

**Conclusion:**

The highly efficient R5 transfer to T_EM _cells suggests that mucosal T cells are an important target for DC-mediated transmission. This may contribute to the initial burst of virus replication that is observed in these cells. T_N _cells, which are the prime target for DC-mediated X4 virus transmission in our study, are considered to inefficiently support HIV-1 replication. Our results thus indicate that DC may play a decisive role in the susceptibility of T_N _cells to X4 tropic HIV-1.

## Background

Several CD4^+ ^T cell subsets can be identified in humans: naïve T cells (T_N_) to mount an immune response to a variety of new antigens, and memory T cells to respond to previously encountered pathogens. T_N _cells preferentially circulate between blood and secondary lymphoid tissues, using high endothelial venules to enter lymph nodes [1]. The memory T cell pool comprises distinct populations of central memory (T_CM_) and effector memory T cells (T_EM_), characterized by distinct homing and effector function [2,3]. Like T_N _cells, T_CM _cells express CCR7 and CD62L, two receptors required for migration to T cell areas of secondary lymphoid tissue. They furthermore have limited effector function, but can proliferate and become T_EM _cells upon secondary stimulation with antigen, and therefore play a role in long term protection. T_EM _cells have lost CCR7 expression, and home to peripheral tissues and sites of inflammation to provide immediate protection against pathogens [2,3]. Consequently, T_N _and T_CM _cells are primarily found in blood and lymphoid tissue, whereas T_EM _cells are enriched in gut, liver and lung. Within the T_EM _cell subset, effector Th1 and Th2 cells are recognized, which are classified by different functional properties based on unique cytokine profiles. Th1 cells produce high levels of IFNγ and TNFβ, which is instrumental in cell-mediated immunity against intracellular pathogens like viruses. Th2 cells secrete a large variety of cytokines (IL-4, IL-5, IL-9 and IL-13) that are crucial for the clearance of parasites, like helminths. Both types of effector cells play a role in the induction of a humoral (antibody) response against different extracellular pathogens [4].

Sexual transmission of HIV-1 involves the crossing of mucosal tissue by the virus, and several studies have shown that one of the very first cell types encountered are intraepithelial and submucosal dendritic cells (DC). Consequently, they have been proposed to facilitate HIV-1 transmission and infection [5-8]. DC are professional antigen presenting cells that sample the environment at sites of pathogen entry. Sentinel immature DC (iDC) develop into mature effector DC (mDC) upon activation by microorganisms or inflammatory signals, and migrate to the draining lymph nodes where they encounter and stimulate naïve Th cells [9,10]. DC are able to capture HIV-1 by a range of receptors, of which the best studied example is DC-SIGN [11]. Subsequent transmission to T cells takes place in lymph nodes via cell-cell contact through an 'infectious synapse' [12]. Additionally, DC can support local virus replication in T cells present in the mucosal tissue [7,8].

An increasing number of studies on HIV-1 and SIV demonstrate that the initial burst of viral replication takes place in CCR5^+ ^CD4^+ ^(effector) memory T cells in the lamina propria of mucosal tissues [13-18]. CCR5 and CXCR4 are the major co-receptors used by HIV-1, with CCR5 being the initial co-receptor used by the virus after transmission. This receptor is primarily expressed on the memory T cell subset and macrophages [19]. Over time, HIV-1 starts to use CXCR4 in some patients, thereby expanding its target cell repertoire to T_N _cells, coinciding with faster disease progression [20,21].

Because DC play an important role in HIV-1 pathogenesis, and T_N_, T_CM _and T_EM _cells have distinct functions and locations in the body, we set out to investigate the contribution of DC in infection of these T cell subsets. We found that CCR5-using (R5) HIV-1 is efficiently transmitted to T_EM _cells but not to T_N _cells. Transmission to T_CM _cells was of intermediate efficiency. Transmission to pure populations of Th1 or Th2 cells, or to an unbiased population containing both types (Th0) was equally efficient. The highly efficient R5 transfer to T_EM _cells suggests that mucosal (T_EM_) cells are an important target for DC-mediated transmission, which may contribute to the observed initial burst of virus replication in these cells. CXCR4-using (X4) HIV-1 could be transmitted to all T cell subsets, due to expression of CXCR4 on all subsets. Surprisingly, X4 HIV-1 was preferentially transmitted to T_N _cells, which are considered to inefficiently replicate X4 HIV-1 [22-24]. This study shows that co-receptor expression is a decisive factor for DC-mediated HIV-1 transmission, and more importantly, that DC may play a crucial role in making T_N _cells susceptible to X4 HIV-1 replication later in infection.

## Results

### T cell subsets differ in susceptibility to DC-mediated transmission of R5 and X4 HIV-1

To investigate whether different CD4^+ ^T cell subsets differ in their susceptibility to DC-mediated HIV-1 transmission, we isolated by live sorting highly purified populations of CD45RA^+ ^CD45RO^- ^naïve T cells (T_N_) and CD45RA^- ^CD45RO^+ ^memory T cells from pure CD4^+ ^T cells. Based on the expression of CCR7, a homing receptor for secondary lymphoid tissue, the memory pool was further divided in CCR7^+ ^central memory T cells (T_CM_) and CCR7^- ^effector memory T cells (T_EM_) [2,3]. We subsequently incubated DC with the R5 virus JR-CSF isolate or the X4 virus LAI isolate for 2 hr, followed by washing steps to remove unbound virus. After addition of the respective T cell subsets, we determined the transmission efficiency by measuring the accumulation of HIV-1 capsid protein p24 (CA-p24) in T cells by FACS. To prevent subsequent rounds of HIV-1 replication after transmission in this single-cycle transmission assay, we added an inhibitor of the viral protease (saquinavir, [25,26]).

**Figure 1:**
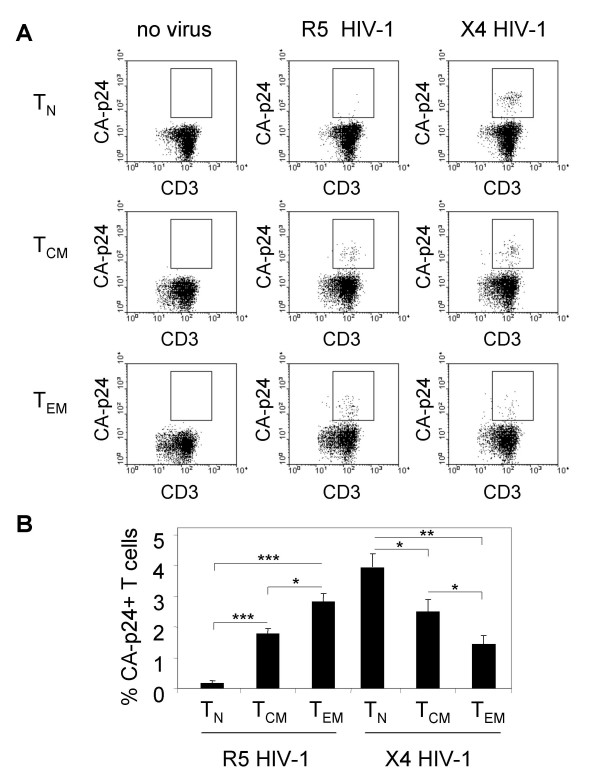
**T cell subsets differ in susceptibility to DC-mediated transmission of R5 and X4 HIV-1**. (A) DC were incubated with R5 or X4 HIV-1, or mock treated, followed by extensive washing to remove unbound virus. DC were subsequently co-cultured with CD4^+ ^naïve T cells (T_N_), central memory T cells (T_CM_) or effector memory T cells (T_EM_) in the presence of saquinavir to prevent spreading infection (single-cycle transmission assay). Two days after transmission, T cells were harvested and stained for CD3 and intracellular CA-p24 to determine the percentage HIV^+ ^T cells. Representative FACS plots are shown. (B) Summary of one representative experiment. Error bars represent standard deviations. * p < 0.05 ; ** p < 0.01; *** p < 0.001.

In a control experiment without HIV-1, no CA-p24 positive CD3^+ ^T cells were scored (Fig. 1A). Addition of R5 HIV-1 resulted in high percentages of CA-p24^+ ^T_EM _cells, and hardly any CA-p24^+ ^T_N _cells (2.9 and 0.1 %, respectively). The transmission to T_CM _cells was of intermediate efficiency (1.9%). With X4 HIV-1, the pattern was reversed: X4 HIV-1 was preferentially transmitted to T_N _cells (4%), then to T_CM _cells (2.2%), and the transmission to T_EM _cells was least efficient (1.4%) (Fig. 1A). Overall, X4 transmission was more efficient than R5 transmission, and could take place to all subsets. For both viruses, the percentage CA-p24^+ ^T cells reached a maximum value 2 days post transmission, and these data are quantified in Fig. 1B. This experiment demonstrates that there is not one exclusive T cell subset that is the preferred target of DC-mediated HIV-1 transmission, but that the efficiency depends on the tropism of the transmitted virus.

### DC-mediated HIV-1 transmission is co-receptor dependent

**Figure 2:**
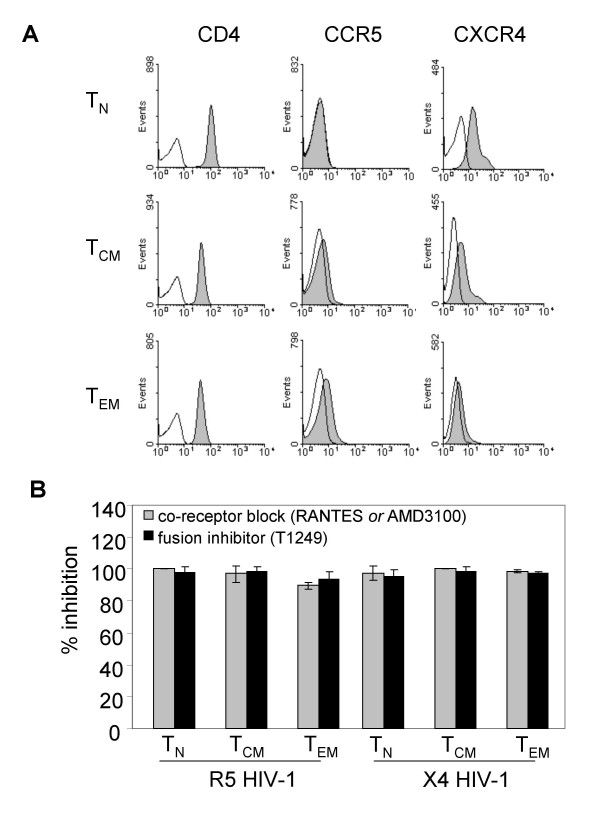
**DC-mediated HIV-1 transmission is co-receptor dependent**. (A) FACS analysis of T_N_, T_CM _and T_EM _cells for CD4 and co-receptors CCR5 and CXCR4. Open histograms represent isotype controls. (B) Transmission inhibition by co-receptor ligands and a fusion inhibitor. A single-cycle transmission assay to T_N_, T_CM _and T_EM _cells was performed with R5 and X4 HIV-1 loaded DC. Prior to co-culture with DC, the T cells were pre-incubated with ligands for CCR5 (RANTES) or CXCR4 (AMD3100) (grey bars) or alternatively, with fusion inhibitor T1249 (black bars). After 2 days, the percentage CA-p24^+ ^T cells was determined by FACS. The percentage inhibition of transmission relative to transmission without inhibitors is indicated on the y-axis. Error bars represent standard deviations.

The different transmission patterns for R5 and X4 HIV-1 prompted us to investigate the co-receptor expression on each T cell subset (Fig. 2A). We found that the level of co-receptor expression for both CCR5 and CXCR4 correlates with the transmission efficiencies depicted in Fig. 1B: CCR5 expression is most pronounced on T_EM _cells, and is undetectable on T_N _cells; CXCR4 is detectable on all subsets, but its expression declines from T_N _cells via T_CM _to T_EM _cells.

To investigate the role of co-receptor expression in DC-mediated HIV-1 transmission, we added the well-described inhibitors RANTES and AMD3100 in the single-cycle transmission assay. These compounds inhibit HIV-1 infection of T cells by blocking the co-receptors CCR5 and CXCR4, respectively [27,28]. Transmission of HIV-1 was completely blocked through the addition of these compounds (Fig. 2B, grey bars). We furthermore could block transmission completely with inhibitor T1249 (Fig. 2B, black bars). This peptide prevents fusion of viral and cellular membranes [29]. Our results thus demonstrate that DC-mediated HIV-1 transmission requires 'regular' infection through CD4 and a co-receptor.

### Method of T cell stimulation determines HIV-1 susceptibility

**Figure 3:**
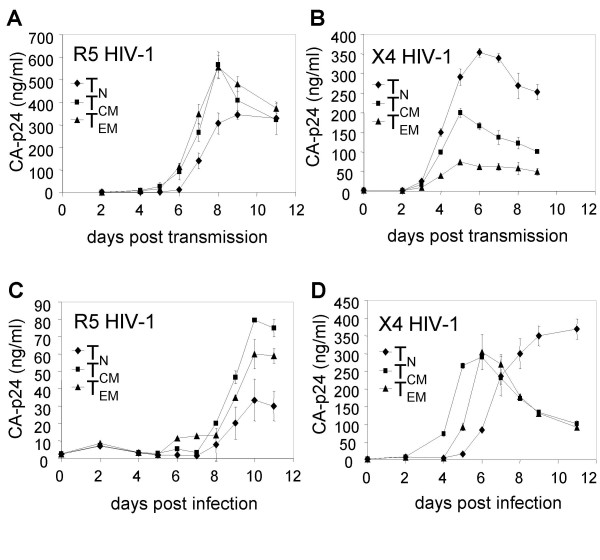
**Spreading infection assay**. Replication of R5 (A) and X4 (B) virus in T_N_, T_CM _and T_EM _cells after DC-mediated HIV-1 transmission. Alternatively, the T cell subsets were stimulated by crosslinking CD3/CD28 with antibodies and infected with R5 (C) or X4 (D) virus. Viral replication was followed by CA-p24 ELISA on the supernatant. Error bars represent standard deviations.

In addition to quantification of the transmission efficiency in a single-cycle transmission assay (Fig. 1 and 2), we followed viral replication after transmission (Fig. 3). In this spreading infection assay, we did not add saquinavir to allow cell-cell spread of newly produced virus. Replication of R5 and X4 HIV-1 in T_N_, T_CM _and T_EM _cells following DC-mediated transmission reflects the results of the single-cycle transmission assay: R5 HIV-1 preferentially replicates in memory T cells, whereas X4 HIV-1 prefers T_N _cells over the memory subsets (Fig. 3A and 3B).

Since this spreading infection assay involves two different steps, *i.e*. transmission and subsequent replication, we also studied R5 and X4 HIV-1 replication in T_N_, T_CM _and T_EM _cells in a DC-independent system. Therefore, cellular proliferation was induced by cross linking of CD3 and CD28 on the T cells with antibodies (Fig. 3C and 3D). As expected, the susceptibility of all T cell subsets to R5 HIV-1 replication was low after CD3/CD28 stimulation. This phenomenon was previously described for CD4^+ ^T cells in general, and is the consequence of CCR5 down regulation and production of natural CCR5 ligands that compete for co-receptor binding [30,31]. But despite this low replication capacity, the pattern of R5 replication was comparable to the replication after DC-mediated transmission of R5 HIV-1: replication was lower in T_N _cells. Surprisingly, X4 replication in T_N _cells was significantly delayed in comparison to T_CM _and T_EM _cells, which does not reflect the enhanced transmission and replication in T_N _cells in the transmission experiments (Fig. 1 and 3B).

**Figure 4:**
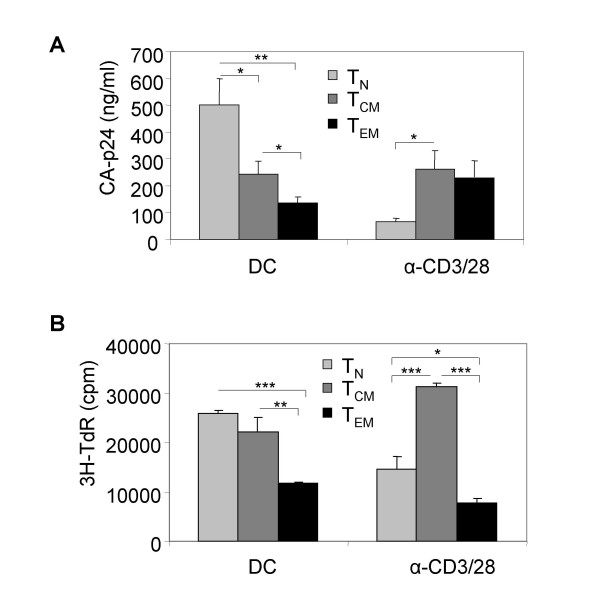
**Method of T cell stimulation determines HIV-1 susceptibility**. (A) Comparison of viral replication in T_N_, T_CM _and T_EM _cells that were stimulated by DC or by CD3/CD28 crosslinking with antibodies. The T cells were stimulated for 4 days, harvested and re-plated before infection with X4 HIV-1. Viral spread was followed by CA-p24 ELISA, of which the results of day 6 are shown. (B) To measure T cell proliferation T_N_, T_CM _or T_EM _cells were incubated with DC or α-CD3/CD28 antibodies and after 4 days, cellular proliferation was determined by ^3^H-thymidine incorporation. Error bars represent standard deviations. * p < 0.05 ; ** p < 0.01; *** p < 0.001.

This discrepancy prompted us to compare HIV-1 replication in T cells stimulated by either DC or α-CD3/CD28 antibodies, without any complicating factors like transmission steps. We therefore stimulated all T cell subsets with DC, or alternatively, with α-CD3/CD28 antibodies and harvested the T cells after 4 days of proliferation. The cells were subsequently infected with X4 HIV-1. DC-stimulated T_N _cells were more susceptible to X4 HIV-1 replication than the memory subsets (Fig. 4A), which reflects the replication after transmission (Fig. 3B). The reverse was observed with α-CD3/CD28 stimulated T cells (Fig. 4A), which is in concordance with the results of Fig. 3D in which the cells were infected immediately after stimulation. This indicates that the enhanced replication of X4 HIV-1 in T_N _cells following DC-mediated transmission, is due to a higher HIV-1 susceptibility. It further demonstrates that crosslinking of CD3 and CD28 by antibodies is not comparable to DC-T cell stimulation, although this crosslinking is considered to mimic DC encounter. The difference between both stimulation methods is further manifested by the proliferative capacity of the T cells, as determined by ^3^H-thymidine incorporation (Fig 4B). The proliferation pattern of the different T cell subsets after DC or α-CD3/CD28 stimulation is clearly not the same.

### DC transmit HIV-1 with equal efficiency to Th1 and Th2 cells, or to an unpolarized population

The T_EM _cell subset can be further divided into effector Th1 and Th2 cells [4]. We generated *in vitro *polarized populations of pure Th1 and Th2 cells, or an unbiased population containing both types (Th0 cells), by culturing purified T_N _cells with or without IL-12 or IL-4, as previously described [32]. We next investigated whether HIV-1 is differently transmitted to these subsets of effector Th1, Th2 or Th0 cells. In addition, we tested different mature DC subsets. Depending on the type of pathogen and tissue factors, immature DC develop into mature effector DC that are specialized to stimulate naïve T cells to develop into IFNγ-producing Th1 cells or IL-4-producing Th2 cells, designated DC1 and DC2 respectively [33]. DC0 induce an unpolarized response (Th0). DC0, DC1 and DC2 were generated by culturing immature DC with maturation factors (MF, IL-1β and TNFα) only (DC0), or MF with either IFNγ (DC1) or prostaglandin E_2 _(DC2) [34].

**Figure 5:**
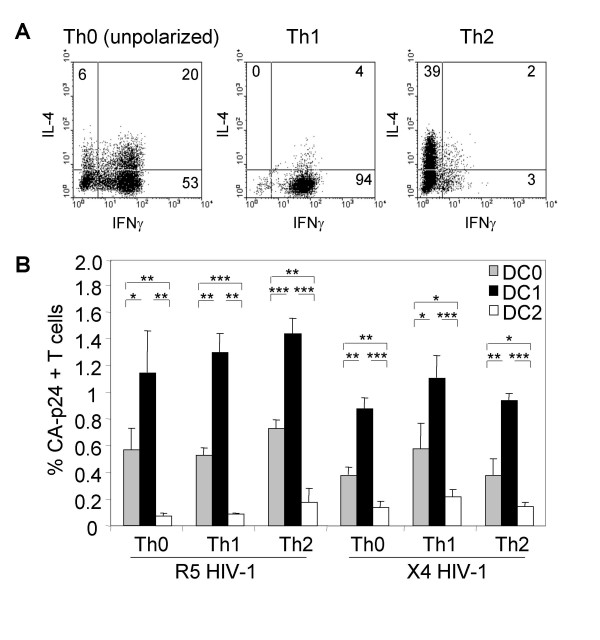
**DC transmit HIV-1 with equal efficiency to Th0, Th1 and Th2 cells**. (A) *In vitro *generated polarized populations of Th1 and Th2 cells, or an unbiased population (Th0), were analyzed for intracellular cytokines IFNγ and IL-4 by FACS. The percentage single and double positive cells is indicated. (B) Th0, Th1 and Th2 cells were co-cultured with R5 or X4 virus-loaded DC in a single-cycle transmission assay to determine the transmission efficiency. Different DC subsets were used: DC1 that stimulate T_N _cells to develop into Th1 cells, DC2 that induce Th2 cells, or DC0 that induce an unpolarized response (Th0). The percentage CA-p24^+ ^T cells was determined by FACS 2 days post transmission. Error bars represent standard deviations. * p < 0.05 ; ** p < 0.01; *** p < 0.001.

The intracellular cytokine profiles of the effector Th cell populations were analyzed by FACS (Fig 5A). The Th1 population consists primarily of IFNγ producers, whereas the Th2 population contains mostly IL-4 producers. The unpolarized Th0 population is composed of both cell types. All T cell subsets expressed similar levels of CCR5 and CXCR4, and proliferated to a comparable extent, as determined by ^3^H incorporation (results not shown).

DC0, DC1 and DC2 were subsequently incubated with R5 and X4 HIV-1, followed by washing and addition of Th0, Th1 and Th2 cells. Two days later, the transmission efficiency was determined in the single-cycle transmission assay (Fig. 5B). Consistent with Fig. 1B, R5 virus was a bit more efficiently transmitted to these polarized T_EM _cells than X4 HIV-1. More importantly, we found no significant differences in HIV-1 transmission efficiency to Th0, Th1 or Th2 cells within one DC subset, *i.e*. a particular DC subset transmits HIV-1 with equal efficiency to Th0, Th1 or Th2 cells. We also did not find a preference of HIV-1 transmission by a DC subset and its corresponding Th type: DC1 was the most efficient HIV-1 transmitter in all cases. The latter was previously demonstrated by us, using unpolarized peripheral blood leukocytes (PBL) and T cell lines [35]. We now show that this also applies to polarized Th subsets.

## Discussion

T_N_, T_CM _and T_EM _cells have distinct functions and locations in the body [1,2], which may have, combined with the differential expression of HIV-1 co-receptors, an impact on HIV-1 transmission and infection. Since DC play an important role in HIV-1 pathogenesis, we studied the DC-mediated transmission of R5 and X4 virus to the different T cell subsets. Although we used only two (well-described) strains of HIV-1, our results suggest that in general R5 HIV-1 is preferentially transmitted to T_EM _cells, whereas DC transmit X4 HIV-1 most efficiently to the T_N _subset.

It is known that R5 viruses are primarily transmitted between individuals and that X4 viruses emerge only later in infection [19,36]. An increasing number of studies on HIV-1 and SIV demonstrate that the initial burst of viral replication takes place in CCR5^+ ^CD4^+ ^(effector) memory T cells in the lamina propria of the mucosa [13-18]. Later in infection, proviral DNA can be isolated from both naïve and memory CD4^+ ^T cells [37,38]. The mechanism responsible for R5 predominance early in infection is not known. One proposed mechanism is the exclusive transport of R5 viruses over the epithelial barrier by epithelial CCR5^+ ^cells [39]. Moreover, DC were proposed to be responsible due to the preferential replication of R5 HIV-1 [40-42], although this R5 replication is not entirely exclusive [43-46]. In addition, DC do not need to be productively infected to transmit HIV-1 to T cells [47,48], and DC can transmit both X4 as R5 HIV-1 to T cells [42]. In fact, we demonstrate in this study that X4 virus is generally transmitted more efficiently than R5 virus. Therefore, DC are probably not the 'gatekeepers' that select R5 viruses, although their role in sexual transmission is a crucial one [7,8].

One of the remaining questions is whether DC either facilitate local HIV-1 replication, or transport the virus to the lymph nodes, or both [7,8,19]. R5 HIV-1 is efficiently transmitted to T_CM _cells (Fig. 1), which are primarily present in lymphoid tissue, and even more efficiently to T_EM _cells, which are abundantly present at sites of viral entry in the mucosa. This suggests that transmission can take place at both locations.

Although X4 HIV-1 is very efficiently transmitted to T_N _cells, X4 virus does not emerge in recently infected HIV patients. Thus, DC-mediated X4 HIV-1 transmission to T cells may not take place following sexual transmission, or may not be a factor of relevance. DC may nonetheless play an important role later in infection (when X4 HIV emerges), e.g. by making T_N _cells susceptible to X4 HIV-1 as we have shown in this study.

We furthermore subdivided T_EM _cells into Th1 and Th2 cells, which did not reveal more differences. DC transmit HIV-1 with equal efficiency to Th1 or Th2 cells, or to an unbiased population containing both types (Th0). Reports on the ability of R5 and X4 virus to replicate in Th0, Th1 or Th2 cells are not univocal [49-52]. Based on our results, the type of T_EM _cell (Th0, 1 or 2) is not of importance for susceptibility to DC-mediated HIV-1 transmission, although the state of activation is an important (though not decisive) factor [53-55]. Furthermore, antigen specific T cells may be preferred [56].

We have shown here that the decisive factor for efficient HIV-1 transmission to the different T cell subsets is co-receptor expression. These HIV-1 transmission results with DC are in concordance with other studies that have shown *in vivo *and *ex vivo *the correlation between differential expression of CCR5 and CXCR4 on naïve and memory T cells and HIV-1 susceptibility [57-59]. We are the first to further divide the memory T cell pool into populations of effector and central memory T cells. We furthermore found that the presence of DC seems to enhance HIV-1 infection and replication, but does not change the pattern of susceptibility. Under certain conditions, no correlation was found between co-receptor expression and HIV-1 susceptibility. When the T cells were stimulated with α-CD3/CD28 antibodies, replication of X4 HIV-1 in T_N _cells was restricted in comparison to the memory subsets. We therefore compared stimulation of T cells by α-CD3/CD28 with stimulation by DC, and found differences in T cell proliferation and X4 susceptibility.

Crosslinking CD3 and CD28 by antibodies is a commonly used laboratory method for T cell stimulation, and mimics T cell activation through triggering of these molecules by DC-bound MHC-II and CD80/86, respectively. However, many more interactions play a role in DC-T cell interaction and stimulation, *e.g*. CD30L-CD30; OX40L-OX40; 41BBL-41BB; CD70-CD27; ICOSL-ICOS; CD40-CD40L and ICAM-1-LFA-1 [10,33,60,61]. Each of these interactions could have an influence on the replication capacity of HIV-1 in T cells, and some of these interactions therefore are the subject of further study. Our results demonstrate that DC play a vital role in priming T_N _cells to become susceptible to HIV-1, and that α-CD3/CD28 stimulation is not a very good model for DC stimulation in the context of HIV-1 studies.

## Conclusion

We have shown that DC transmit R5 and X4 HIV-1 with different efficiencies to T_N_, T_CM _and T_EM _cells, and that this correlates with co-receptor expression of the different T cell subsets. The highly efficient transmission of R5 HIV-1 to T_EM _cells, which are abundantly present at sites of viral entry, may contribute to the observed burst of viral replication in these cells after HIV-1 infection. Later on in infection, DC may play an important role in the replication of X4 HIV-1 in T_N _cells.

## Materials and methods

### Generation of monocyte-derived dendritic cells

Peripheral blood mononuclear cells (PBMC) were isolated by density centrifugation on Lymphoprep (Nycomed, Torshov, Norway). Subsequently, PBMC were layered on a Percoll gradient (Pharmacia, Uppsala, Sweden) with three density layers (1.076, 1.059, and 1.045 g/ml). The light fraction with predominantly monocytes was collected, washed, and seeded in 24-well culture plates (Costar, Cambridge, MA, USA) at a density of 5 × 10^5 ^cells per well. After 60 min at 37°C, non-adherent cells were removed, and adherent cells were cultured to obtain immature DC in Iscove's modified Dulbecco's medium (IMDM; Life Technologies Ltd., Paisley, United Kingdom) with gentamicin (86 μg/ml; Duchefa, Haarlem, The Netherlands) and 10% fetal calf serum (HyClone, Logan, UT, USA), supplemented with GM-CSF (500 U/ml; Schering-Plough, Uden, The Netherlands) and IL-4 (250 U/ml; Strathmann Biotec AG, Hannover, Germany). At day 3, the culture medium with supplements was refreshed. At day 6, maturation was induced by culturing the DC with maturation factors only (MF; IL-1β (10 ng/ml) and TNFα(50 ng/ml); Strathmann Biotec AG), or MF with either IFNγ (1000 U/ml; Strathmann Biotec AG), or prostaglandin E_2 _(10^-6 ^M; Sigma-Aldrich, St. Louis, MO), see results for more details [34]. After two days, mature CD14^- ^CD1b^+ ^CD83^+ ^DC were obtained. All subsequent tests were performed after harvesting and extensive washing of the cells to remove all factors. Mature DC were analysed for the expression of cell surface molecules on a FACScan (BD Biosciences, San Jose, CA, USA). Mouse anti-human mAbs were used against the following molecules: CD14 (BD Biosciences), CD1b (Diaclone, Besançon, France), CD83 (Immunotech, Marseille, France) and ICAM-1 (CD54) (Pelicluster, Sanquin, Amsterdam, The Netherlands). All mAb incubations were followed by incubation with FITC-conjugated goat F(ab')_2 _anti-mouse IgG and IgM (Jackson ImmunoResearch Laboratories, West Grove, PA, USA).

### CD4^+ ^T cells

Naïve and memory T cells were live sorted from pure CD4^+ ^T cells on a FACS ARIA (BD Biosciences). The following mouse-anti-human antibodies were used: CD45RA-FITC (Coulter, Hialeah, FL, USA), CD45RO-APC (BD Biosciences), CD4-PE-Cy7 (BD Biosciences). Rat-anti-human CCR7 (BD Biosciences) incubation was followed by biotin-rabbit-anti-rat (Zymed Laboratories Inc., San Francisco, CA, USA) and streptavidin-PerCp-Cy5.5 (BD Biosciences) incubation. CD4^+ ^CD45RA^+ ^CD45R0^- ^cells were considered naïve T cells (T_N_). CD4^+ ^CD45RA^- ^CD45R0^+ ^cells (the memory population) was separated into central memory (T_CM_) (CCR7^+^) and effector memory (T_EM_) (CCR7^-^) cells, according to the classification described by Sallusto *et al *[2]. Polarized Th1 and Th2 cells, and an unpolarized population containing both types (Th0 cells) were generated from purified T_N _cells as previously described [32]. In short, T_N _cells (10^5^/200 μl) were stimulated with immobilized α-CD3 (CLB-T3/3; 1 μg/ml) and α-CD28 (CLB-CD28/1; 2 μg/ml) (both from Sanquin, Amsterdam, The Netherlands) and cultured for 10 days in the absence (Th0) or presence of IL-12 (100 U/ml; a gift from Dr. M. K. Gately, Hoffma-La Roche) or IL-4 (1000 U/ml) for Th1 and Th2 cells respectively. To generate fully polarized Th cells, the cells were restimulated with PHA (10 μg/ml; Difco, Detroit, MI, USA) and 3000 rad-irradiated feeder cells (PBMC of two unrelated donors and EBV-B cells (JY cells)) in the presence of IL-4 for Th0 cells; IL-4 neutralizing antibodies (CLB_IL-4/6, Sanquin) plus IL-12 for Th1 cells; and IL-12 neutralizing antibodies (U-CyTech, Utrecht, the Netherlands) plus IL-4 for Th2 cells. All T cells were cultured in IMDM with 10% FCS, gentamycin and IL-2 (Cetus, Emeryville, CA, USA). During co-culture with DC, *Staphylococcus enterotoxin B *(SEB; Sigma-Aldrich; final concentration, 10 pg/ml) was added. α-CD3/CD28 stimulation of T cells for viral replication experiments was done with mouse mAb to human CD28 (CLB-CD28/1) and human CD3 (CLB-T3/4E-1XE, Sanquin).

### Cytokine production by polarized Th cells

12 days after the second stimulation round, resting T cells were restimulated with PMA (10 ng/ml) and ionomycin (1 μg/ml) for 6 hr, the last 4.5 hr in the presence of Brefeldin A (10 μg/ml) (all Sigma-Aldrich). Cells were fixed in 2% PFA, permeabilized with 0.5% saponin (Sigma-Aldrich), and stained with anti-IFNγ -FITC and anti-IL4-PE (both BD Biosciences). Cells were then analysed by FACS.

### Virus stocks

C33A cervix carcinoma cells were transfected using calcium phosphate with 5 μg of the molecular clone of CXCR4-using HIV-1 LAI or CCR5-using HIV-1 JR-CSF. The virus containing supernatant was harvested 3 days post transfection, filtered and stored at -80°C. The concentration of virus was determined by CA-p24 ELISA. C33A cells were maintained in Dulbecco's Modified Eagle's Medium (DMEM) (Invitrogen, Breda, the Netherlands), supplemented with 10% FCS, 2 mM sodium pyruvate, 10 mM HEPES, 2 mM L-glutamine, penicillin (100 U/ml) (Sigma-Aldrich) and streptomycin (100 μg/ml; Invitrogen).

### HIV transmission assay and CA-p24 measurement

Fully matured DC (IFNγ/MF if indicated otherwise) were incubated in a 96-well-plate (45 × 10^3 ^DC/100 μl/well) with HIV-1 (15 ng CA-p24/well) for 2 hr at 37°C. The DC were washed with PBS after centrifugation at 400 × g to remove unbound virus. Washing was repeated 2 times, followed by addition of 50 × 10^3 ^T_N_, T_CM _or T_EM _cells. In some experiments, T1249 (250 ng/ml; Trimeris, Durham, NC, USA), RANTES (500 ng/ml, R&D Systems, Abingdon, UK) or AM3100 (10 μg/ml, Sigma-Aldrich) was added. The latter two were pre-incubated with the T cells for 30 min at 37°C. Prior to addition to DC, the T cells were analyzed by FACS with the following mouse anti-human antibodies: CD4-PE, CCR5-PE and CXCR4-PE (all BD Biosciences). Viral replication after transmission was followed by measuring CA-p24 in the culture supernatant by ELISA. To determine intracellular CA-p24 in the single-cycle transmission assay, saquinavir (Roche, London, UK at 0.2 μM) was added to prevent cell-to-cell spread of newly produced virions. After 48 hr, the T cells were harvested and stained with FITC-labeled CD3 (BD Biosciences), followed by fixation with 4% PFA and washing with washing buffer (PBS with 2 mM EDTA and 0.5% BSA). Fixated cells were then washed with perm/wash buffer (BD Biosciences), and incubated with PE-labeled CA-p24 (KC57-RD1, Coulter) followed by washing with successively perm/wash- and washing buffer. Cells were then analysed by FACS.

### T cell proliferation

Fully matured DC (45 × 10^3 ^DC/well) were incubated in a 96-well-plate with T_N_, T_CM_, T_EM _cells, or polarized Th cells (50 × 10^3 ^T cells/well) in a final volume of 200 μl. After 2 days, cell proliferation was assessed by the incorporation of [^3^H]-TdR after a pulse with 13 KBq/well during the last 16 hr of the co-culture, as measured by scintillation spectroscopy. Alternatively, T_N_, T_CM _or T_EM _cells were stimulated with α-CD3/CD28 antibodies, followed by the [^3^H]-TdR pulse 2 days later.

### Statistical analysis

Data were analysed for statistical significance (GraphPad InStat, Inc, San Diego, CA, USA) using ANOVA. A *p *value < 0.05 was considered to be significant.

## Competing interests

The author(s) declare that they have no competing interests.

## Authors' contributions

FG designed the study, performed the experiments and wrote the paper; TMMVC participated in the proliferation assays, JHNS participated in the isolation of the T cell subsets, BB helped to write the manuscript, ECDJ designed the study and helped to write the manuscript.
